# Influence of New Technology in Dental Care: A Public Health Perspective

**DOI:** 10.3390/ijerph20075364

**Published:** 2023-04-03

**Authors:** Antonio Gracco, Alberto De Stefani, Giovanni Bruno

**Affiliations:** 1 Department of Neuroscience, School of Dentistry, University of Padova, 35122 Padova, Italy; antonio.gracco@unipd.it (A.G.);; 2 Department of Pharmacological Sciences, University of Padova, 35122 Padova, Italy; 3 Department of Industrial Engineering, University Tor Vergata, 00133 Rome, Italy

The advent of new technology has caused significant changes in the field of dentistry, enabling dentists and orthodontists to provide more efficient and effective treatments to their patients. From digital X-rays to 3D printing, technological advancements have transformed the way dental care is delivered, making it more accurate, safe, and patient-friendly.

The application of technology in dentistry is not just limited to diagnosis and treatment. It has also made dental care more accessible and affordable, especially for underserved populations. In this editorial article, we discuss the influence of new technology in dental care from a public health perspective.

The use of digital X-rays has revolutionized the way dentists diagnose oral health problems. In digital radiography, sensors are used to capture images of hard and soft tissues, which can be instantly viewed on a computer screen. This eliminates the need for traditional film X-rays, which require longer processing times and expose patients to higher radiation doses.

Digital impressions represent another technological advancement that has transformed the field of dentistry. Instead of using messy and uncomfortable analogical impressions, dentists can now take digital impressions of a patient’s dental arch using an intraoral scanner. This technology allows for more accurate and precise impressions, reducing the need for repeat appointments and increasing patient comfort. In a study included in this Special Issue, the authors compared the accuracy of four leading intraoral scanners for full-arch implant rehabilitation [1].

Three-dimensional printing is another technological advancement that has had a significant impact on dental care. It has revolutionized the way dentists create crowns, bridges, and other dental prosthetic restorations. With 3D printing, dentists can create highly detailed models of a patient’s teeth, which can be used to design and create customized dental restorations. This technology has reduced the time required to create dental restorations, making the process more efficient and cost-effective [2].

In addition to the impact on diagnosis and treatments, new technology in dental care has also had a significant impact on the education and training of dental students. Dental schools are now incorporating these new technologies into their courses, allowing students to gain hands-on experience. This provides students with a more comprehensive understanding of the field and prepares them to deliver the highest quality of care to their future patients. The use of simulation technology enables students to practice procedures in a safe and controlled environment, allowing them to develop their skills without the risk of harming patients. Overall, the integration of new technology into dental education has enhanced the learning experience for students, preparing them for a successful and rewarding career in the field [3].

New technology has improved patient outcomes by making dental care safer and more effective. One example of this is the use of lasers in dental procedures. Lasers can be used to remove decay, reshape gums, and even perform root canals. They are less invasive than traditional dental tools, which can lead to less bleeding and discomfort for the patient.

In addition, the use of digital tools in treatment planning has improved patient outcomes. Another technological advancement currently implemented in dental care is the use of facial scanners, such as the Vectra system [4]. This system uses 3D imaging technology to capture images of a patient’s face and mouth, allowing dentists to create a more detailed and customized treatment plan. The system can be used to simulate the effects of various dental procedures on a patient’s facial structure, enabling dentists to make more informed decisions about treatment options. The use of facial scanners has improved the accuracy of treatment planning, leading to better patient outcomes and higher levels of patient satisfaction. Facial scans can be aligned with CBCT and intraoral scans to create highly detailed treatment plans that consider a patient’s unique oral anatomy. This leads to more accurate and effective treatment, resulting in better patient outcomes.

New technology has had a significant impact on dental care, making it more efficient, safe, and accessible. From digital X-rays to 3D printing, technological advancements have transformed the way dental care is delivered, improving patient outcomes and also making care more affordable for underserved populations. As technology continues to advance, it is important for dental professionals to stay up to date with the latest developments to provide the best possible care to their patients. From a public health perspective, the impact of new technology on dental care has been overwhelmingly positive, and it is likely that this trend will continue in the years to come.

## References

[1] Di Fiore A., Graiff L., Savio G., Granata S., Basilicata M., Bollero P., Meneghello R. (2022). Investigation of the Accuracy of Four Intraoral Scanners in Mandibular Full-Arch Digital Implant Impression: A Comparative In Vitro Study. Int. J. Environ. Res. Public Health.

[2] Unkovskiy A., Huettig F., Kraemer-Fernandez P., Spintzyk S. (2021). Multi-Material 3D Printing of a Customized Sports Mouth Guard: Proof-of-Concept Clinical Case. Int. J. Environ. Res. Public Health.

[3] Panpisut P., Doungkom P., Padunglappisit C., Romalee W., Suksudaj N. (2023). Assessment of 3D-Printed Tooth Containing Simulated Deep Caries Lesions for Practicing Selective Caries Removal: A Pilot Study. Int. J. Environ. Res. Public Health.

[4] De Stefani A., Barone M., Hatami Alamdari S., Barjami A., Baciliero U., Apolloni F., Gracco A., Bruno G. (2022). Validation of Vectra 3D Imaging Systems: A Review. Int. J. Environ. Res. Public Health.

